# Quantitation of alpha-linolenic acid elongation to eicosapentaenoic and docosahexaenoic acid as affected by the ratio of n6/n3 fatty acids

**DOI:** 10.1186/1743-7075-6-8

**Published:** 2009-02-19

**Authors:** Kerstin Harnack, Gaby Andersen, Veronika Somoza

**Affiliations:** 1 Deutsche Forschungsanstalt für Lebensmittelchemie, Lichtenbergstrasse 4, 85748 Garching, Germany; 2 University of Wisconsin, Department of Food Science, 103 Babcock Hall, 1605 Linden Drive, Madison, WI 53706-1565, USA

## Abstract

**Background:**

Conversion of linoleic acid (LA) and alpha-linolenic acid (ALA) to their higher chain homologues in humans depends on the ratio of ingested n6 and n3 fatty acids.

**Design and methods:**

In order to determine the most effective ratio with regard to the conversion of ALA to eicosapentaenoic acid (EPA) and docosahexaenoic acid (DHA), human hepatoma cells were incubated with varying ratios of [^13^C] labeled linoleic acid ([^13^C]LA)- and alpha-linolenic acid ([^13^C]ALA)-methylesters. Regulative cellular signal transduction pathways involved were studied by determinations of transcript levels of the genes encoding delta-5 desaturase (D5D) and delta-6 desaturase (D6D), peroxisome proliferator-activated receptor alpha (PPARα) and sterol regulatory element binding protein 1c (SREBP-1c). Mitogen-activated protein kinase kinase 1 (MEK1) and mitogen-activated protein kinase kinase kinase 1 (MEKK1) were also examined.

**Results:**

Maximum conversion was observed in cells incubated with the mixture of [^13^C]LA/[^13^C]ALA at a ratio of 1:1, where 0.7% and 17% of the recovered [^13^C]ALA was converted to DHA and EPA, respectively. Furthermore, differential regulation of enzymes involved in the conversion at the transcript level, dependent on the ratio of administered n6 to n3 fatty acids in human hepatocytes was demonstrated.

**Conclusion:**

Formation of EPA and DHA was highest at an administered LA/ALA ratio of 1:1, although gene expression of PPARα, SREBP-1c and D5D involved in ALA elongation were higher in the presence of ALA solely. Also, our findings suggest that a diet-induced enhancement of the cell membrane content of highly unsaturated fatty acids is only possible up to a certain level.

## Introduction

Long chain polyunsaturated fatty acids (LCPUFA) of the n6 and n3 families as well as their precursors are essential dietary components for mammalian species [1]. LCPUFAs appear to be a necessary integral part of membrane phospholipids for optimal biological function particularly in specialized cells and tissues such as brain, retina, testes, heart, liver and kidneys [2]. The constitutive properties of PUFA in biological membranes generally are regarded in terms of their contributions to the fluidity or integrity of membrane bilayer structures [3,4]. Increasing evidence for competitive interactions between PUFA of the n6 and n3 fatty acid families at the level of formation and action of eicosanoids, thromboxanes, prostacyclins, prostaglandins, and leukotrienes appear to relate directly to potential benefits attributed to an eligible dietary balance of n3 and n6 fatty acids [5]. Within the common Western diet, ALA represents the quantitatively dominant fatty acid of the n3 series. Daily ALA uptake averages 1.5 g, whereas the average daily uptake of the corresponding n6 fatty acid LA is about 10 times higher [6].

With this 10:1 ratio of dietary n6 to n3 LCPUFA, the formation of pro-inflammatory/aggregatory eicosanoids from LA is favored over those from n3 fatty acids showing anti-inflammatory/aggregatory effects. Controlling the synthesis of n3 LCPUFA, such as EPA and DHA from ALA, and the respective eicosanoids by consumption of the optimized ratio of n6 to n3 LCPUFA is, therefore, of particular nutritional interest.

In addition, LCPUFA and their derivatives are of increasing interest as modulators of gene expression. They are reported for example to be ligands of the nuclear transcription factors peroxisome proliferator-activated receptors (PPARs) [7] and suppressors of sterol regulatory element binding proteins (SREBP) [8]. Regulating the degradation of fatty acids by mitochondrial as well as by peroxisomal and microsomal fatty acid oxidation, PPARα plays a central role in fatty acid homeostasis (reviewed in Ref. [9]). SREBP-1 isoforms on the other hand are more active in regulating hepatic synthesis of fatty acids [10]. Both, PPARs and SREBPs are reported to regulate expression of delta-5-desaturase (D5D) and delta-6-desaturase (D6D) [11,12]. SREBPs as well as PPARs are, in addition to their ligand-induced regulation activity, substrates for several kinases such as mitogen-activated protein kinases (MAPK) [13,14]. Furthermore, ligands for the PPAR receptor family such as the essential fatty acids linoleic (LA, 18:2-n6) and alpha-linolenic (ALA, 18:3-n3) acid have recently been shown to induce MAPK phosphorylation [15,16].

LA and ALA are converted to their longer chain homologues by a combination of subsequent reactions, involving Δ^6 ^desaturation, elongation and Δ^5 ^desaturation. Since members of the n6 and n3 families compete for the corresponding enzyme systems [17-19], the conversion of e.g. LA or ALA into their longer chain homologues is significantly influenced by the composition of dietary fats [20,21]. In this context, whether incorporation of n3 fatty acids into cell membranes and their bioconversion into higher homologues depends on either the absolute amount of dietary n3 fatty acids or rather on the ratio of ingested n6/n3 fatty acids has been controversially discussed.

In a human intervention trial by Liou et al. 2007 [22], diets containing LA/ALA ratios of 10:1 or 4:1 were administered to healthy men. The different LA/ALA ratios were adjusted by altering the LA content in the diet (high-LA or low-LA), while the ALA content was maintained constant. As a result, the ALA as well as the EPA concentration of plasma phospholipids in the low-LA group (4:1 ratio) increased compared to the high-LA group (LA/ALA ratio 10:1), although the ALA amount administered was equal in both groups. This result clearly shows that lowering dietary LA intake increases EPA in plasma phospholipids, indicating the rate of conversion of ALA to EPA being dependent on the ratio of n3/n6 fatty acids. In contrast to that, Goyens et al. 2006 [23] postulated that bioconversion of ALA in humans is influenced by the absolute amounts of dietary ALA and LA and not by their ratio. In this study, healthy volunteers were randomly divided into 3 experimental groups: 1) high ratio LA group (LA/ALA = 19:1), 2) low ratio low-LA group (LA/ALA = 7:1) and 3) low ratio high-ALA group (LA/ALA = 7:1). Within the latter two groups, the 7:1 ratio was adjusted by lowering the LA content or increasing the ALA content of the diet respectively, compared to the diet of the high ratio LA group. In addition to the diet, multiple tracer bolus doses of [U-^13^C]ALA were administered to the subjects to estimate ALA conversion by compartmental modeling. Calculations derived from this study confirmed the hypothesis that conversion of ALA is rather influenced by the absolute amounts of dietary ALA and LA than by the ratio of ALA/LA. However, analyses of plasma phospholipid fatty acid composition at the end of this dietary intervention showed that concentrations of ALA and EPA within the two low ratio groups (LA/ALA = 7:1) were significantly higher compared to the high ratio LA group. However, there was no difference between the ALA and EPA plasma phospholipid contents of the two low ratio groups. These results, on the other hand, support the hypothesis that incorporation and bioconversion of ALA are rather influenced by the ratio of dietary LA/ALA. However, no final conclusion can be drawn from the data available concerning the question, whether the conversion is rather dependent on the absolute amount of fatty acids than on the ratio of n6/n3 fatty acids. Furthermore, quantitative data on the conversion of n3 LCPUFA as affected by the ratio of n6 to n3 fatty acids are scarce. Therefore, the objective of our study was to quantify the conversion of [^13^C]alpha-linolenic acid to eicosapentaenoic acid and docosahexaenoic acid in the presence of varying n6/n3 fatty acid ratios in the hepatoma cell line HepG2. In addition, the transcript levels of genes encoding for enzymes involved in the fatty acid conversion were also examined after incubation of the cells with different ratios of n6/n3 LCPUFAs.

## Experimental procedures

### Cell culture conditions

HepG2 cells were obtained from Dept. of Human and Animal Cell Cultures (DSMZ), Braunschweig, Germany. The cells were grown in T-75 cell culture flasks (Sarstedt, Germany) in 15 ml Dulbecco's modified Eagle's medium (DMEM) containing 20% fetal calf serum (FCS), 2% glutamine and 2% penicillin/streptomycin solution (Sigma). Cells were maintained at 37°C in a humidified atmosphere of 95% air/5% CO_2 _and were subcultured every 7 days. For the experiments carried out, cells were transferred to T-25 cell culture flasks (Sarstedt, Germany) at a density of 5 × 10^6 ^cells. After 24 hrs, the medium was replaced with 8 ml DMEM without FCS. After another 24 hrs, mixtures of [^13^C] labeled LA/ALA (suspended by sonication in sterile bovine serum albumin, Fraction V, Sigma) were added to give a final fatty acid concentration of 100 μM. Ratios of LA/ALA were 9:1, 4:1, 1:1, 1:0 and 0:1. Cells were incubated for 24 hrs at 37°C and the exposure in each case was carried out as a quadruplicate determination. At the end of the exposure process, cells were harvested by adding 1 ml of accutase (Sigma). The cell culture medium was collected separately for determination of [^13^C]LA and [^13^C]ALA content. After harvesting, cells were washed with 2 ml of FCS free DMEM and centrifuged at 500 × g. After adding 1 ml of sodium chloride solution (0.9%; w/v) to the cell pellet, cells were stored at -75°C.

### Lipid extraction and fatty acid analysis

Lipids were extracted from the cells by a modified Folch extraction [24], with the addition of 100 μl of internal standard (1 mg/ml of tetracosanoic acid and 3 mg/ml heptadecanoic acid, dissolved in dichlormethane:methanol, 2:1, v:v) to each sample. The extraction procedure was followed by saponification and methylation of the fatty acids according to an AOAC method [25]. Fatty acid composition was determined via HRGC-CI/-MS [26]. Determination of absolute contents of the several fatty acids was carried out by external calibration.

### Cell culture experiments

For gene expression experiments, cells were pretreated as described above. Within the scope of kinetic experiments, cells were exposed to 100 μM pure ALA-methylester for 3, 6, 12 and 24 hrs, respectively, to find out the point of maximal gene expression of D5D, D6D, PPARα, SREBP-1c, MEK1 and MEKK1 via real time PCR (MX 3000P, Stratagene).

For dose response experiments, HepG2 cells were exposed to mixtures of LA-/ALA-methylester at ratios of 4:1, 1:1, 1:0 and 0:1. Here, the 9:1 ratio was not tested since there was no difference between the 9:1 and the 4:1 mixture with regard to [^13^C]ALA-incorporation and the formation of [^13^C]EPA and [^13^C]DHA.

Depending on the results of kinetic experiments, expression of the several aforesaid target genes was determined after 3, 6 or 12 hrs. After incubation, cells were harvested as described earlier and RNA was isolated with RNeasy mini kit^® ^(Qiagen). cDNA was synthesizd using StrataSkript^® ^cDNA synthesis kit (Stratagene) according to manufacturer's instructions. For amplification of gene transcripts during the realtime PCR reactions, validated primer assays from Qiagen were used according the manufacturer's protocol. Human transferrin receptor was chosen as house keeping gene. Brilliant^® ^SYBR^® ^Green (Stratagene) was used for detection of amplified gene products during the PCR reaction.

### Statistical analysis

Data were analyzed with SPSS for WINDOWS 14.0 (SPSS Inc, Chicago, IL). Data were tested for normal distribution using the Kolmogorov-Smirnov test. All data are expressed as mean ± SEM. Statistical significance of results was assessed using two-tailed Student's t-test for non-paired samples. A p-value of less than 0.05 was considered statistically significant.

## Results

### Experiments with non-labeled fatty acids to identify experimental conditions of putative maximum elongation rates

The time-dependent effects regarding the incorporation of ALA-methylester and a resulting increase in EPA and DHA concentrations were investigated. For this purpose, Hep-G2 cells were incubated with 100 μM ALA-methylester over periods of 0 h, 6 h, 12 h, 24 h, 48 h and 72 h. Subsequently, dose response experiments were performed with ALA-methylester at concentrations of 10 μM, 100 μM and 1000 μM and an exposure time of 24 hrs. Highest incorporation of ALA methylester and the most pronounced increase in EPA and DHA were analyzed after a 24 h incubation period in the presence of 100 μM ALA-methylester (data not shown).

### Experiments with labeled fatty acids to investigate ALA elongation and desaturation

HepG2 cells were exposed to varying mixtures of [^13^C] labeled ALA and LA. Whereas the total concentration of LCPUFA administered was the same for all experiments (100 μM), the ratios of [^13^C]ALA to [^13^C]LA tested were 0:1, 1:1, 4:1, 9:1 and 1:0.

Total recoveries of [^13^C]LA and [^13^C]ALA as well as the amounts of [^13^C]EPA, [^13^C]DHA and [^13^C]AA at the different ratios of [^13^C]LA and [^13^C]ALA are given in Table 1.

**Table 1:** Recovery of [^13^C]LA or [^13^C]ALA after exposure of HepG2 cells to [^13^C]LA-/[^13^C]ALA-methylester at ratios of 0:1, 1:1, 4:1, 9:1 and 1:0 for 24 hours. ^1^not detected, LOD: 0.13 ng/μl.

	**LA/ALA 0:1**	**LA/ALA 1:1**	**LA/ALA 4:1**	**LA/ALA 9:1**	**LA/ALA 1:0**
^13^**C LA administered [μmol]**	**0**	**50**	**80**	**90**	**100**
**cell culture medium [μmol]**	n.d.^1^	**7.1 **± 0.75	**3.8 **± 0.35	**4.5 **± 0.14	**7.0 **± 0.37
**cells [μmol]**	n.d.^1^	**37.6 **± 1.96	**66.0 **± 2.51	**75.3 **± 1.80	**74.6 **± 2.97
**percent administered [%]**	-	**83**	**84**	**75**	**75**

^13^**C ALA administered [μmol]**	**100**	**50**	**20**	**10**	**0**
**cell culture medium [μmol]**	**9.0 **± 1.2	**4.7 **± 0.27	**1.3 **± 0.11	**2.1 **± 0.11	n.d.^1^
**cells [μmol]**	**65.3 **± 3.90	**32.6 **± 2.15	**16.41 **± 0.31	**6.90 **± 0.17	n.d.^1^
**Percent administered [%]**	**65**	**65**	**82**	**69**	-

Cellular recovery of [^13^C]LA was highest (75.3 μmol or 74.6 μmol) in cells incubated with the mixture of a n6 to n3 ratio of 9:1 or with pure LA, respectively. Of the administered amount of [^13^C]LA, the highest percentage recovery as [^13^C]LA was detected in cells which were exposed to the mixtures of 4:1 (83%) or 9:1 (84%), respectively, whereas in cells exposed to the 1:1 mixture or to pure LA, merely 75% of the [^13^C]LA applied to the cells were recovered. The amount of [^13^C]AA quantified was highest in cells exposed to the mixtures at n6 to n3 ratios of 4:1 (5.7 μmol) or 9:1 (6.3 μmol), respectively (Table 1 and 2).

**Table 2:** Quantification of [^13^C]AA, [^13^C]EPA and [^13^C]DHA after exposure of HepG2 cells to [^13^C] LA-/[^13^C]ALA-methylester at ratios of 0:1, 1:1, 4:1, 9:1 and 1:0 for 24 hours.

	**LA/ALA 0:1**	**LA/ALA 1:1**	**LA/ALA 4:1**	**LA/ALA 9:1**	**LA/ALA 1:0 **
^13^**C LA administered [μmol]**	-	**50**	**80**	**90**	**100**
^13^**C AA [μmol]**	-	**3.4 **± 0.04	**5.7 **± 0.32	**6.3 **± 0.21	**3.3 **± 0.27

^13^**C ALA administered [μmol]**	**100**	**50**	**20**	**10**	-
^13^**C EPA [μmol]**	**10.8 **± 0.51	**5.7 **± 0.19	**1.5 **± 0.05	**1.4 **± 0.04	-
^13^**C DHA [μmol]**	**0.18 **± 0.02	**0.48 **± 0.01	**0.11 **± 0.01	**0.04 **± 0.004	-

**Figure 1:**
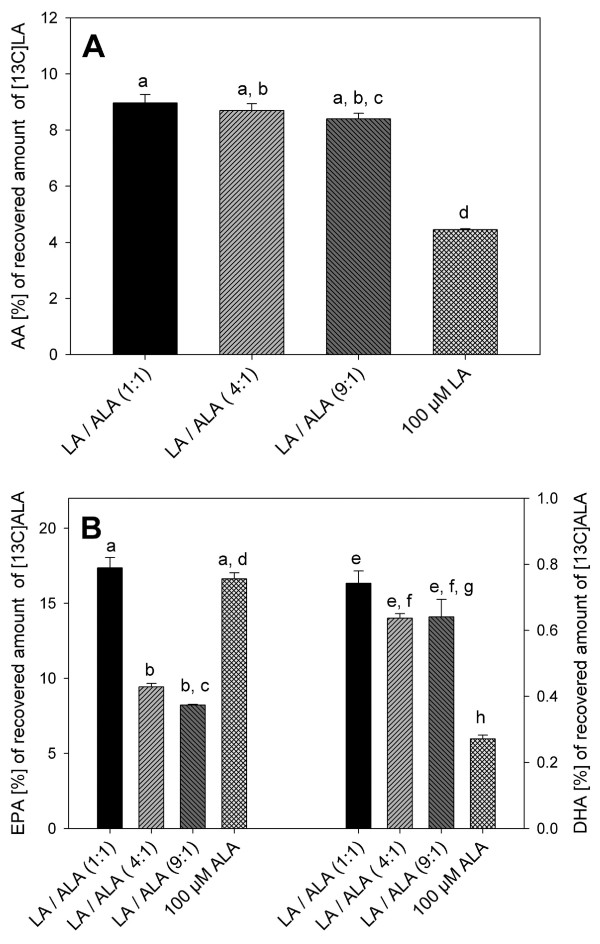
**Percentage conversion of [^13^C]LA and [^13^C]ALA into [^13^C]AA and [^13^C]EPA/[^13^C] after exposure of HepG2 cells to [^13^C]LA-/[^13^C]ALA-methylester at ratios of 0:1, 1:1, 4:1, 9:1 and 1:0**. Data are expressed as mean ± SEM. Bars without a common letter are statistically significant different (*: p ≤ 0.05).

Of the recovered cellular amounts of [^13^C]LA, the amount of [^13^C]AA quantified was equally high in those cells which were incubated with [^13^C]LA/[^13^C]ALA at ratios of 1:1, 4:1 and 9:1, whereas treatment of the cells with pure [^13^C]LA resulted in a significantly lower content of [^13^C]AA (Fig. 1A). Total cellular recovery of [^13^C]LA ranging between 85 and 96% (data not shown) was calculated from the sum of quantified [^13^C]AA and [^13^C]LA in the cell culture medium and in the cells.

As well as incorporation of [^13^C]LA, effective incorporation of [^13^C]ALA increased with escalating amounts of administered [^13^C]ALA (Table 1). Percentage cellular recovery of [^13^C]ALA, relating to its administered amount, was 65% in cells which were exposed to pure ALA or to the mixture at a ratio of 1:1, and 69% in cells incubated with the 9:1 mixture. The highest recovery of [^13^C]ALA (82%) was documented in cells which were exposed o the 4:1 fatty acid mixture of [^13^C]LA and [^13^C]ALA.

The content of [^13^C]EPA also increased with ascending amounts of administered [^13^C]ALA and was the highest in cells exposed to pure [^13^C]ALA (10.8 μmol). On the other hand regarding cells which were exposed to mixtures of [^13^C]LA/[^13^C]ALA, the amount of [^13^C]DHA was highest (0.48 μmol) at a ratio of 1:1.

Of the recovered quantity of [^13^C]ALA, the content of [^13^C]EPA was highest in cells incubated with pure [^13^C]ALA and a mixture of [^13^C]LA/[^13^C]ALA at a ratio of 1:1 (Fig. 1B). In those cells, the amount of [^13^C]EPA in each case was 17% of the recovered quantity of [^13^C]ALA. Moreover, the highest percentage amount of DHA (0.7% of recovered [^13^C]ALA) was formed in cells incubated with [^13^C]LA/[^13^C]ALA at a ratio of 1:1.

Total recoveries of [^13^C]ALA (as a sum of cellular recovered [^13^C]ALA and [^13^C]EPA or [^13^C]DHA, respectively and the [^13^C]ALA remaining in the cell culture medium) ranged between 86% (0:1 and 1:1 mixture) and 97% (5:1 mixture) (data not shown).

### Gene expression experiments

#### Kinetic experiments

**Figure 2:**
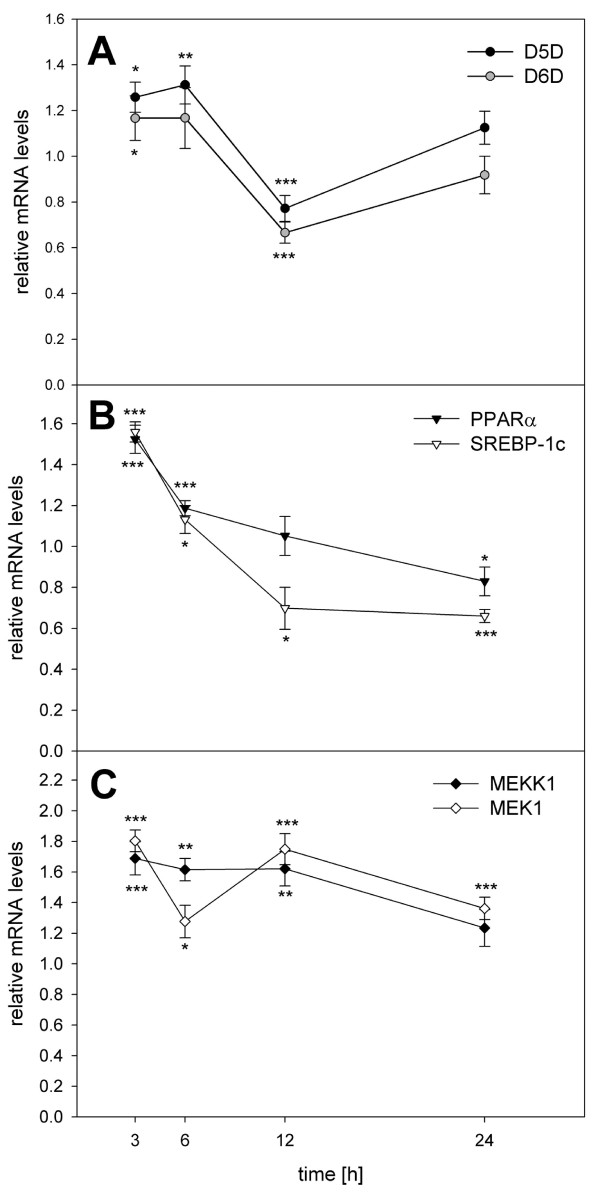
**Kinetics experiments – relative mRNA levels of D5D, D6D, PPARα, SREBP-1c, MEKK1 and MEK1 after 24 hrs exposure of HEPG2 cells to ALA-methylester, relating to non-exposed control cells (mRNA levels set as 1)**. Data are expressed as mean ± SEM. Asterisks indicate a significant difference of the values compared to control cells (*: p ≤ 0.05 **: p ≤ 0.01, ***: p ≤ 0.001).

Since enhanced amounts of [^13^C]EPA and [^13^C]DHA were detected in HepG2 cells after incubation with [^13^C]ALA, we wanted to investigate whether this conversion is associated with enhanced expression of genes encoding for proteins related to lipid metabolism. Therefore, HepG2 cells were incubated with 100 μM of ALA methylester for 3, 6, 12 and 24 hrs and the transcript levels of the genes encoding for D5D, D6D, PPARα, SREBP-1c, MEKK1 and MEK1 were determined via Real-time-PCR (Fig. 2). Maximum mRNA levels of the D5D and the D6D gene were detected after 3 and 6 hrs of cells' treatment. The mRNA levels of the genes encoding for nuclear receptor proteins PPARα and SREBP-1c were highest after an incubation period of 3 hrs. The quantity of mRNA of the kinases MEKK1-gene and MEK1-gene was the highest in those cells which were incubated with ALA-methylester for 3 and 12 hrs, respectively.

#### Dose response experiments

**Figure 3:**
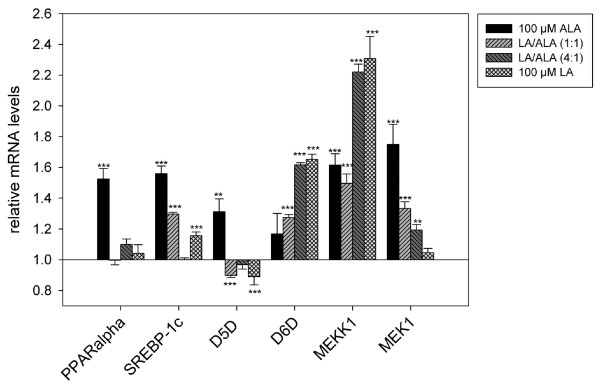
**Dose response experiments – relative mRNA levels of D5D, D6D, PPARα, SREBP-1c, MEKK1 and MEK1 after exposure of HEPG2 cells to LA-/ALA-methylester at ratios of 0:1, 1:0, 1:1 and 4:1, relating to non-exposed control cells (mRNA levels set as 1)**. Data are expressed as mean ± SEM. Asterisks indicate a significant difference of the values compared to control cells (**: p ≤ 0.01, ***: p ≤ 0.001).

The kinetic experiments revealed that after 3 hrs of incubating HepG2 cells with 100 μM ALA methylester, the transcript levels of the investigated genes reached their maximum. At this point in time, a dose-response experiment was carried out in order to investigate the influence of different mixtures of LA/ALA on the mRNA levels of the genes encoding for D5D, D6D, PPARα, SREBP-1c, MEKK1 and MEK1 (Fig. 3).

Compared to non-exposed control cells, the transcript level of the MEKK1 gene was significantly increased in any of the experiments, with incubation of the cells with pure LA and the 4:1 mixture of LA-/ALA-methylester caused the most considerable effects. In contrast, the transcript level of the MEK1 gene was mainly affected by ALA and not by LA. With respect to the experiments in which HepG2 cells were exposed to pure ALA-methylester, an incubation of the cells with the 1:1 and 4:1 mixture of LA-/ALA-methylester induced a significant increase (up to 35%) of the mRNA level of the gene encoding for MEK1.

Transcript levels of genes encoding for PPARα and D5D, respectively, were only increased after exposure of HepG2 cells to pure ALA-methylester. Compared to non-exposed cells, mRNA levels of these genes were about 30–50% higher in cells that were incubated with pure ALA-methylester.

As well as PPARα and D5D, the transcript level of the SREBP 1c-gene was significantly increased after incubation with pure ALA-methylester. The relative mRNA level was about 75% higher than in non-exposed cells. Furthermore, an exposure of HepG2 cells to pure LA-methylester and the 1:1 mixture of LA-/ALA-methylester, respectively, caused a significant increase in SREBP-1c gene transcript levels of up to 30%.

## Discussion

In the here presented study, a human hepatoma cell line (HepG2) was used as a model system to investigate the impact of varying ratios of n6/n3 fatty acids on the conversion of alpha-linolenic acid to eicosapentaenoic acid and docosahexaenoic acid. After exposure of HepG2 cells to different ratios of [^13^C] labeled 18:2-n6 and 18:3-n3, those fatty acids were quantified in the cell matrix. Furthermore, conversion to the corresponding homologues was demonstrated. At this, rates of incorporation as well as the extent of conversion differed depending on the ratio of administered fatty acids.

### Incorporation of [^13^C]LA and [^13^C]ALA in hepatocytes

Effective incorporated quantities of [^13^C]ALA as well as [^13^C]LA increased with increasing administered amounts of these fatty acids. Percentage quantities of cellular recovered [^13^C]LA or [^13^C]ALA, relating to the administered amount, however were lower in cells exposed to 100 μM of a single fatty acid ([^13^C]LA or [^13^C]ALA) than in cells exposed to mixtures of [^13^C]LA and [^13^C]ALA. We assume that these findings may result from a limited ability of the cells to incorporate PUFAs beyond a certain level.

A study of Robinson et al. [27] confirms this hypothesis. In this study, the modulating effect of dietary fish oil and of n3 fatty acid methylesters on spleen phospholipid fatty acid composition was investigated. Female NZB/W mice were fed diets containing 3.6 or 10% of EPA or DHA ethyl ester (EPA-E, DHA-E), respectively. EPA or DHA levels in phospholipids increased with increasing quantities of dietary EPA-E or DHA-E, respectively, but the total phospholipid n3 levels were highest in animals to which the lowest dose of EPA-E or DHA-E was administered. Furthermore, Venkatraman et al. [28] investigated the existence of a dietary maximum level for incorporation of n3 fatty acids into membrane phospholipids of weanling rats. In this study, animals were fed a diet which provided increasing amounts of C20 and C22 n3 fatty acids such as C20:5-n3 and C22:6-n3, and after 4 weeks of feeding, the fatty acid composition of membrane phospholipids of liver nuclear envelopes was analyzed. An increase in n3 dietary fatty acid administration from 1.1% to 5% of total fatty acids resulted in an increase in 20:5-n3 membrane phospholipid content. But further rise of dietary n3 fatty acids did not lead to an increased incorporation of 20:5-n3 into membrane phospholipids.

### Conversion of [^13^C]LA and [^13^C]ALA to their longer chain homologues

In addition to the incorporation, the administered fatty acids have also been converted to their longer chain homologues, as we could clearly demonstrate by using labeled [^13^C]LA and [^13^C]ALA. As well as rates of incorporation, the extent of conversion was affected by the ratio of administered fatty acids. Regarding the conversion of [^13^C]18:2-n6 to 20:4-n6, it was remarkable that the amount of AA formed was significantly lower in cells exposed to 100 μM [^13^C]LA than in cells which were incubated with mixtures of [^13^C]LA and [^13^C]ALA. However, when comparing the percentage rates of conversion of [^13^C]LA and [^13^C]ALA to AA and EPA/DHA, respectively, the conversion of [^13^C]ALA to EPA/DHA was higher than the conversion of [^13^C]LA to AA. This effect might be attributed to a differential effect of fatty acids of the n6 series and the n3 series on desaturase activity and/or gene expression. Since suppression of D5D and D6D activities by dietary unsaturated fatty acids was shown to be associated with decreased D5D and D6D mRNA levels [29,30], we investigated whether these transcript levels were altered after 6 hrs of incubating the HepG2 cells with either LA and ALA or mixtures of both fatty acids.

Interestingly, exposure of HepG2 cells to 100 μM LA caused a marked (p < 0.001) increase of the transcript level of the D6D gene, whereas the exposure to 100 μM ALA did not alter the transcript levels of the D6D gene. In contrast, mRNA level of the gene encoding for D5D was significantly (p < 0.001) decreased only by exposure to 100 μM of pure LA, relating to non-exposed control cells. Administration of LA alone or as part of the mixture led to a strong decrease of the transcript level of the D5D gene, relating to non-exposed cells. Since both, Δ^6^- and Δ^5^-desaturation reactions, were shown to be rate-limiting for conversion of LA or ALA to their longer chain homologues, we assume that formation of 20:4-n6 in cells incubated with 100 μM LA was limited by reduced D5D gene expression.

Despite of high amounts of EPA formed in cells exposed to 100 μM [^13^C]ALA, conversion to DHA was significantly (p < 0.001) lower in those cells compared to cells incubated with lower doses of [^13^C]ALA in the form of [^13^C]LA/[^13^C]ALA mixtures.

Similar results regarding the conversion of EPA to DHA were described by Robinson et al. [27]. After feeding diets containing 3%, 6% or 10% of EPA ethyl ester (EPA-E) to female mice there were no increases in spleen phospholipid DHA levels in animals fed either the 10% or the 6% EPA-E diet. Only administration of the 3% EPA-E diet was associated with an elevation of DHA levels in phospholipids. According to these findings, we assume that administration of n3 PUFAs beyond a certain level suppresses formation of DHA from EPA. However, the general extent of conversion from ALA to EPA and DHA in men was reported to be about 8% for EPA and less than 0.05% for DHA [6]. The conversion rates in our experiments were about 16% for EPA and about 0.7% for DHA, after incubation of the cells with an [^13^C]LA/[^13^C]ALA mixture of 1:1. This result gives rise to the possibility of shifting the conversion of ALA to EPA and DHA to higher rates by adjustment of the ratio of n6/n3 fatty acids.

### Partitioning of LA and ALA towards β-oxidation

Since the total recovery (calculated by addition of total cellular recovery and recovery within cell culture medium) of the administered fatty acids was below 100%, it seems likely that oxidation of LA and ALA also took place since both fatty acids are known to be substrates for β-oxidation. This hypothesis of partial degradation of [^13^C]LA and [^13^C]ALA via β-oxidation in our experiments is supported by results by a study of Mayes et al. [20], who administered [^13^C] labeled ALA to preterm infants. Appearance of ^13^CO_2 _in breath, which was determined as a marker of ALA partitioning towards β-oxidation, ranged from 7.6 to 19%. In our experiments, the amount of non-recovered [^13^C]ALA or [^13^C]LA ranged between 3 and 14%, respectively. Therefore, we assume that this portion of [^13^C]ALA or [^13^C]LA was used for β-oxidation since, in addition, molecular masses of non-enzymatically generated oxidation products were not detected by HRGC-CI/-MS (data not shown).

Generally, genes of fatty acid oxidation are induced by the peroxisome proliferators-activated receptor alpha (PPARα). Furthermore, fatty acids such as alpha-linolenic acid are considered natural ligands of PPARα because they were shown to bind and activate PPARα *in vitro *[30].

The administration of 100 μM pure ALA alone effectuated a marked induction of PPARα (p < 0.001) in our experiments. Also, total recovery of [^13^C]ALA in cells exposed to pure [^13^C]ALA was considerably lower, compared to cells incubated with any of the mixtures of [^13^C]LA and [^13^C]ALA. Hence, we presume that [^13^C]ALA was preferentially used as substrate for PPARα-mediated β-oxidation in cells incubated with pure [^13^C]ALA. However, the amount of ^13^CO_2 _was not analyzed in this experiment.

### Effect of LA and ALA on lipogenesis

The transcription factor SREBP-1c plays a pivotal role in the dietary regulation of most hepatic lipogenic genes [31], including the expression of D5D and D6D [13] and its primary role is assumed to be the regulation of highly polyunsaturated fatty (PUFA) acid supply for membrane phospholipids [32]. Expression of SREBP-1c genes was shown to be down-regulated by dietary PUFA [33]. In contrast to this, the here presented results demonstrate that, after 3 hrs of exposure time, the transcript level of SREBP-1c gene was significantly (p < 0.001) induced by exposition of HepG2 cells to LA or ALA at a concentration of 100 μM and the mixture of LA and ALA at a ratio of 1:1, respectively. This result may imply a dose and time dependent SREBP-1c gene regulation which has to be proven in future studies.

Besides SREBP-1c, peroxisome proliferators such as alpha-linolenic acid paradoxically are identified as activators of D6D and probably D5D gene transcription [11-13]. He et al. [12] hypothesized hat this up-regulation of gene transcription might be a compensatory response to increased demand in PUFAs caused by induction of fatty acid oxidation.

An intracellular activation of the mitogen-activated (MAP) kinase cascade by ectopic expression of these upstream activators had a direct stimulatory effect on the transcriptional activity of SREBP-1c via phosphorylation of MAP kinases ERK1 and ERK2 [34]. Hence, PPARα is also reported as a substrate for several kinases such as ERK1 and ERK2 [14,35], in addition to ligand-induced regulation, transcriptional activity of PPARα may increase via phosphorylation of certain domains of the receptor protein [36]. In this work, the transcript levels of the MEKK1 and MEK1 genes were markedly (p < 0.001) increased after the cells' exposure to LA, ALA or mixtures of both. In clear contrast to the transcript level of MEKK1 gene, the transcript levels of the MEK1 gene were lower, the higher the LA-content in the mixture was. However, we did not measure an activation of the transcriptional activities of either PPARα or SREBP-1c via MAP kinase phosphorylation. But the enhanced transcript level could indicate an induction of the respective pathway, which may has led to the observed induction of D6D and D5D. Further investigations with regard to receptor-ligand-binding and state of phosphorylation of PPARα or SREBP-1c receptor proteins in the presence of defined ration of n6/n3 PUFAs are necessary to elucidate the mechanisms of gene expression.

## Conclusion

Our investigations show that exposure of human hepatocytes to different mixtures of LA and ALA affected transcript levels of a portfolio of genes encoding regulating proteins involved in several stages of fatty acid metabolism. This effect strongly depends on the ratio of n6/n3 fatty acids, indicating the importance of ingesting an appropriate amount of fatty acids, but also an appropriate ratio of n6 and n3 fatty acids. This is further confirmed by the fact that maximum conversion of LA to AA was measured only in the presence of ALA. Omitting ALA seems to result in higher oxidation rates as indicated by high transcript levels of PPARα. Additionally, the n6/n3 ratio strongly influenced the elongation of ALA to EPA and DHA as well as the transcript levels of D5D and D6D.

However, from a nutritional perspective, our findings suggest that a diet-induced enhancement of the cell membrane content of highly unsaturated fatty acids is only possible up to a certain level. Increasing the administered quantity of labeled alpha-linolenic acid resulted in a reduced effectiveness of [^13^C]ALA incorporation. Percentage incorporation of [^13^C]ALA was maximal after exposure of [^13^C]LA/[^13^C]ALA at a ratio of 4:1 to HepG2 cells.

With regard to the elongation of ALA to EPA and DHA, our results clearly indicate the efficiency of altering the dietary ratio of LA to ALA in favor of ALA by increasing the concentration of n3 fatty acids in human hepatocytes. At this, administration of LA/ALA at a ratio of 1:1 led to the highest formation of LCPUFAs of the n3 series.

## Abbreviations

**[^13^C]LA**: ^13^C labelled linoleic acid; **[^13^C]ALA**: ^13^C labelled alpha linolenic acid; **AA**: arachidonic acid; **ALA**: alpha-linolenic acid; **D5D**: delta-5 desaturase; **D6D**. delta-6 desaturase; **DHA**: docosahexaenoic acid; **DMEM**: Dulbecco's modified Eagle's medium; **EPA**: eicosapentaenoic acid; **FCS**: fetal calf serum; **HUFA**: highly unsaturated fatty acids; **LA**: linoleic acid; **LCPUFA**: long-chain polyunsaturated fatty acids; **MAPK**: mitogen-activated protein kinase; **MEK1**: mitogen-activated protein kinase kinase 1; **MEKK1**: mitogen- activated protein kinase kinase kinase 1; **PCR**: polymerase chain reaction; **PPARα**: peroxisome proliferator-activated receptor alpha; **SREBP**: sterol regulatory element binding protein

## Competing interests

The authors declare that they have no competing interests.

## Authors' contributions

KH and GA performed the analysis of the biochemical data, participated in the interpretation of the results and helped draft the manuscript. VS designed the study and assisted with data interpretation and manuscript preparation.
